# Improved CNN with BiLSTM model for early melanoma and skin lesion classification

**DOI:** 10.3389/frai.2026.1808770

**Published:** 2026-04-28

**Authors:** S. Rajeshkumar, Chiranji Lal Chowdhary

**Affiliations:** School of Computer Science Engineering and Information Systems, Vellore Institute of Technology, Vellore, TN, India

**Keywords:** BiLSTM, CNN, computer-aided diagnosis, deep learning, HAM10000, melanoma, skin cancer

## Abstract

Melanoma contributes to approximately 1% of all skin cancer cases but accounts for the majority of the deaths due to its aggressive behavior and high metastatic potential. Traditional diagnostic methods, including dermoscopy via the ABCDE rule, tend to be subjective and often lead to significant interobserver error, with accuracy rates generally ranging from 65% to 80%. This variability creates an urgent need for improved diagnostic tools that can enhance patient outcomes and reduce the burden on healthcare systems. Recent advancements in deep learning have transformed skin lesion classification. While CNNs excel at extracting spatial features, they often struggle to capture long-range contextual dependencies, an important factor in distinguishing visually similar lesions. Standard sequential models, such as Long Short-Term Memory (LSTM) networks, help address some of these gaps but have the drawback of being unidirectional. To overcome these limitations, this paper proposes a hybrid CNN-Bidirectional LSTM (BiLSTM) model. An all-encompassing preprocessing pipeline was developed using the HAM10000 dataset, which contains 10,015 dermoscopic images categorized into seven diagnostic labels. This pipeline includes noise reduction, lesion segmentation, data augmentation, normalization, and feature extraction, each aligned with the ABCDE rule. The CNN extracts hierarchical spatial information, while the subsequent BiLSTM layer learns contextual dependencies in both directions, allowing for a more nuanced description of lesions. The CNN-BiLSTM model with the suggested architecture has shown to be effective in the domain of automated classification of skin lesions, with an accuracy of 94.82%, a precision of 93.76%, a recall of 92.94% and an F1-score of 93.35% on the HAM10000 dataset, demonstrating its effectiveness in automated skin lesion classification with an inference time of only 80 ms per image, it is suitable for real-time clinical use. These results reinforce the clinical prospects of bidirectional contextual modeling in early melanoma detection and may have applications in broader computer-aided diagnosis systems.

## 1 Introduction

Skin cancer is one of the most prevalent malignancies all over the world, where the rates of incidence are on a sharp increasing trend over the decades. Clinically, skin cancer is categorized into melanoma and non-melanoma, basal cell carcinoma (BCC), and squamous cell carcinoma (SCC) (World Health Organization, 2023). Though the incidence of melanoma makes up only approximately 1000 cases in the total skin cancer cases, it causes most deaths associated with skin cancer because it metastasizes easily, and in most cases, it develops quickly without early diagnosis (Abohashish et al., 2025). The most important environmental risk factor is prolonged exposure to ultraviolet (UV) radiation, which contributes to approximately 90% age of non-melanoma cases as well as approximately 86% of melanoma cases in the whole world (Tschandl et al., 2018). Even though there is an increased level of living awareness and preventive actions, the cases of melanoma keep increasing. This reiterates the dire need to have solutions to early-diagnostic problems that are reliable, scalable, and with a high degree of accuracy.

Dermoscopy nowadays is the clinical gold standard of noninvasive assessment of skin lesions. The method improves the visualization of structures in the subsurface and assists in the diagnosis according to the thoroughly known criteria of the ABCDE Asymmetry, Border irregularity, Color variation, Diameter, and Evolution. Although dermoscopy has a greater accuracy in diagnosis than naked-eye inspection, it is still subjective in nature and mostly relies on the experience of the clinician. The accuracy of the diagnosis is reported to be 65% to 80%, and the inter-observer variability is high (Brinker et al., 2019). The visual aspect of the similarity of the benign and malignant lesions will result in false positivity and unwarranted biopsies, and the presence of the early stages of melanoma will go undetected, preventing timely treatment and reducing the probability of survival. These shortcomings have prompted the creation of objective computer-aided diagnosis (CAD) systems to help clinicians in the evaluation of lesions.

In early CAD methods, the use of traditional machine-learning methods, including k-nearest neighbors, decision trees, and support-vector machines, with manually designed features, including color, texture, and shape descriptors, was used (Alwakid et al., 2022). Despite moderate successes, these methods performed poorly due to low robustness as well as generalization when presented with real-life variations that are in imaging (LeCun et al., 2015). With the introduction of deep learning [most prominently, Convolutional Neural Networks (CNNs)], it became possible to perform end-to-end hierarchical feature learning directly on dermoscopic images and perform the task of skin-lesion analysis better than previous (Esteva et al., 2017). The models based on CNN soon became a new paradigm, and multiple studies have shown that the models achieve the level of dermatologists or even higher results in the task of melanoma classification. Later developments, such as transfer learning, the ensemble approach, and architecture optimization, further enhanced accuracy and resistance (Pacheco and Krohling, 2020). With these advantages, traditional CNN designs have the structural trade-off of not being able to capture long-range contextual dependencies due to the local receptive fields of the design. Such diagnostic cues as global asymmetry, lesion-wide border irregularities, and large-scale color transitions tend to be missed in pure CNN models because they tend to be more than a single convolutional window (Xie et al., 2020).

Recent developments in deep learning have made a big breakthrough in the field of automated skin lesion classification. Deep convolutional neural networks (DCNNs) have shown high efficacy in dermoscopic image analysis given the fact that they have the capability of automatically discovering hierarchical features representations using large datasets. Transformer-based architectures have also attracted interest in the medical image analysis in recent years. ViT models have demonstrated appeal in that they can capture global contextual relationships in a medical image using self-attention processes (Nguyen et al., 2024; Ong et al., 2024). These models enable the extraction of long-range dependencies across image regions, which can be useful in complex visual pattern recognition (Himel et al., 2024). Moreover, efficacious model scaling strategy has enabled EfficientNet-based architectures to reach high classification accuracy in dermoscopic image datasets, by balancing network depth, width and resolution, respectively, all at once and simultaneously, compared to traditional network scaling strategies with fewer scales and less capacity to achieve the same accuracy (Alruwaili and Mohamed, 2025; Do et al., 2024). Recent works have shown that EfficientNet models are effective in such benchmark datasets as ISIC and HAM10000, among others, when applied to melanoma and skin-related problems, respectively, as observed (Unnisa et al., 2025; Theerthagiri, 2026). In spite of such developments, issues like class imbalance, variability of domains and even limited interpretability still act as major obstacles to successful clinical implementation. In a bid to resolve these issues, hybrid deep learning architectures that incorporate convolutional feature extraction with sequential modeling techniques have proved to be a good solution. This paper is driven by these problems, so a hybrid CNN-BiLSTM system will be proposed to generate automated skin lesion classification. Spatial features of dermoscopic images are extracted using CNN component, whereas the BiLSTM network takes into account the contextual links between extracted features representations. The suggested framework will enhance the performance of classification and be computationally efficient to be used in practical medical systems.

To address these limitations, researchers have proposed hybrid CNN-Recurrent Neural Network (RNN) architectures, particularly those incorporating Long Short-Term Memory (LSTM) units (Greff et al., 2017). In these models, CNNs extract spatial features, while LSTMs capture sequential relationships between the features, thereby enhancing contextual representation (Das et al., 2022). Several studies have demonstrated that CNN-LSTM models improve sensitivity and recall in melanoma detection compared to standalone CNNs. Still, traditional LSTMs only process sequences in a single temporal direction, restricting the model's ability to capture the full context. To overcome this, BiLSTM networks operate both forward and backwards on sequences, so each element benefits from complete contextual information (Reddy et al., 2023). As a result, hybrid CNN-BiLSTM models have shown superior performance in many medical imaging tasks by effectively integrating localized spatial learning with global contextual modeling. Furthermore, adding attention mechanisms to CNN-BiLSTM architectures further improves both classification capabilities and interpretability by highlighting diagnostically relevant lesion locations (Zhang and Wang, 2023).

**Figure 1:**
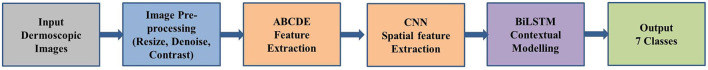
General workflow of the proposed skin lesion recognition framework.

HAM10000 is a dataset of 10,015 dermoscopic images (seven categories of diagnoses), which is considered by many researchers to be a benchmark for assessing hybrid architectures because of its variety and variability in practice. Although there have been major improvements, there are a number of challenges that have remained. Clinical adoption is hampered by severe class imbalance, image artifacts and noise, overfitting due to a small amount of annotated data, as well as the inability to interpret the model. To overcome these problems, solid hybrid frameworks are needed to find a balance between diagnostic accuracy, sensitivity, interpretability, and computational efficiency. With such gaps in place, this work suggests a strong hybrid CNN-BiLSTM model to detect melanoma and classify skin lesions into multiple classes using the HAM10000 dataset. The offered methodology involves extensive pre-processing, two-way contextual modeling, and clinically focused evaluation indicators to increase the reliability of the diagnosis (Codella et al., 2019). Comprehensive experimental analyses indicate that the suggested framework has greater performance than baseline CNN and CNN-LSTM models. It has a high overall accuracy and decreases the false negative rates of melanoma. These findings indicate that CNN-BiLSTM frameworks have the potential to be useful and clinically viable CAD systems to detect skin cancer at early stages. The rest of this paper is structured in the following way: Section 2 gives a review of related work on skin lesion classification; Section 3 outlines the proposed CNN-BiLSTM methodology; Section 4 provides experimental results and discussion; and Section 5 ends with the conclusion about future directions. Figure 1 represents the pipeline, which includes dermoscopic image acquisition, preprocessing, lesion segmentation, ABCDE feature extraction, CNN-based spatial feature learning, BiLSTM-based contextual modeling, and final classification of skin lesion types.

### 1.1 Contributions of this study

Even though CNN-BiLSTM networks have already been used in the medical image analysis field, the current work has a few methodological advances that are designed specifically to be applied in the classification of dermoscopic skin lesions. The principal contributions provided in the work are summarized as follows:

- A hybrid CNN-BiLSTM model implementing the characteristics of spaces (morphological), and sequence-based features (contextual) of dermoscopic images pathology.
- Dermatology-inspired features of an ABCDE rule-based model are integrated with deep CNN embeddings to prevent a lack of diagnostic interpretability and improve the performance of the classifier.
- A training approach to reduce the problem of class imbalance in the HAM10000 dataset through the methods of class weighting and data augmentation.
- Extensive comparison of the proposed architecture and a set of deep learning baselines to show that the proposed architecture is effective in automated skin lesions classification.

The proposed skin lesion classifier is based on CNN, unlike traditional CNN-based skin lesion classifiers. The method combines CNN feature extraction and a BiLSTM module to learn feature embeddings that capture contextual relationships between learned feature embeddings. This mixed design enables the model to acquire spatial as well as sequential dependencies, enhancing the performance of classification on dermoscopic images. In addition, the model includes domain-inspired. Enhance the interpretability with ABCDE dermatology.

## 2 Related work

Computer-aided diagnosis (CAD) systems that are used to classify skin lesions have changed considerably in the last ten years. It has transitioned from conventional handcrafted feature methods to highly sophisticated hybrid deep learning methods. This section summarizes the major contributions of the literature based on CNN, CNN-LSTM, and CNN-BiLSTM deep learning techniques to classify multiclass and melanoma in skin lesions.

### 2.1 Conventional machine learning methods

The initial automated systems of skin lesion classification were based on manual feature extraction and classical machine learning models, including the use of k-nearest neighbors (KNN), decision trees (DT), and support vector machines (SVM). These models in general employed color histograms, texture features based on gray-level co-occurrence matrices (GLCM), and geometric shape features within lesion morphology (Alwakid et al., 2022). Ballerini et al. (2013) developed a hierarchical KNN classifier; the scheme combined color and texture, with moderate classification performance in controlled laboratory settings. Likewise, Viknesh et al. (2019) used SVM classifiers on the texture features based on GLCM, which showed a better performance over the baseline methods. However, the hand-made solutions were weak with respect to real-world scenarios, such as changes in illumination, hair covering, and color variation based on the devices. Oliveira et al. (2020). have tried to integrate the local binary patterns, which are handcrafted, with the deep features that have been extracted with InceptionV3. They were highly accurate and needed much preprocessing and were not very scalable. In general, despite the fact that these methods established some foundational benchmarks, their dependence on manual feature engineering constrained generalization and clinical applicability.

### 2.2 CNN-based deep learning models

The introduction of CNN resulted in a discontinuity in the study of skin lesions as end-to-end learning of hierarchical feature representations from raw dermoscopic images became possible (LeCun et al., 2015). An iconic study, conducted by Esteva et al. (2017), confirmed that deep CNN models were capable of comparable diagnostic performance to trained dermatologists, confirming the clinical promise of deep learning in dermatology. The latter research was followed by investigations into transfer learning and ensemble techniques to address the problem of data scarcity (Litjens et al., 2017). To obtain better robustness under various datasets, Mahbod et al. (2020) used ensemble learning to combine several pretrained CNN structures. Pacheco and Krohling (2020) evaluated systematically the fine-tuned CNN architecture with better generalization than conventional machine learning models. Moura et al. (2019) also included knowledge of dermatological domains by incorporating the ABCD rule into CNN pipelines except Edge, which have high accuracy and are more interpretable. In spite of these developments, the pure CNN models are still constrained by the local receptive fields that limit them from detecting long-distance contextual dependencies. International diagnostic hints like lesion-wide asymmetry, protracted border abnormalities, and smooth colouration shifts are usually poorly modeled using single CNN designs (Shaik et al., 2025).

CNNs are the most popular automated skin lesion classification method. CNN-based models can automatically extract hierarchical feature representations using dermoscopic pictures, and they do not require handcrafted features that have always been employed in the analysis of medical images. VGGNet, ResNet and DenseNet are popular architectures that have been extensively used on dermoscopic image classification problems, and have performed well on publicly-available datasets like ISIC and HAM10000. Recent works have delved into deeper convolutional architectures to enhance accuracy of classification and computational efficiency. The use of models based on EfficientNets has become popular in the classification of skin lesions because they can achieve scaling of network depth, width, and resolution at the same time, as shown by Tan and others in 2019 (Do et al., 2024). These models have been found to perform well in the context of melanoma detection and analysis of dermoscopic images tasks (Alruwaili and Mohamed, 2025).

### 2.3 Transformer-based approaches

Recently, transformer architecture has become an effective computer vision and medical image analysis tool. Vision Transformers (ViT) are also self-attention-based models that capture contextual information in images by being distant to enable models to understand the relationships between distant locations in dermoscopic images (Nguyen et al., 2024). It has also been suggested to implement hybrid CNN transformer architectures that would provide the strengths of convolutional feature extraction and attention-based contextual modeling in a hybrid design (Ong et al., 2024). Moreover, Swin Transformer and other frameworks with transformers have shown performance potentials in medical imaging, enhancing feature representations and global visual patterns capture, among other things, which make them useful in medical imaging tasks (Ramkumar et al., 2024).

### 2.4 Hybrid CNN-LSTM architectures

Hybrid deep learning models are a type of neural network which combines the advantages of more than one to improve the classification. Convolutional networks are efficient in the extraction of spatial features whereas the recurrent neural networks like Long Short-Term Memory (LSTM) and Bidirectional LSTM (BiLSTM) are effective in the extraction of sequential dependencies within feature representations. The use of CNNs with recurrent architectures in dermoscopic image analysis has been studied in order to enhance contextual feature learning. BiLSTM networks are also very practical in that they work in both forward and backwards directions to ensure that the model captures dependencies in the feature sequences. Nonetheless, the hybrid CNNBiLSTM classifiers are not fully studied in the context of dermoscopic image recognition.

In order to overcome the contextual drawbacks of CNNs, hybrid CNN-LSTM models were proposed to include sequential dependency modeling. Within these architectures, CNNs learn spatial properties, whereas long-term relationships between representations of features are learned by Long Short-Term Memory (LSTM) networks, which can be used to model long-term dependencies (Hochreiter and Schmidhuber, 1997). Abohashish et al. (2025) have proposed a CNN-LSTM model, tested on the HAM10000 data, which has better recall and sensitivity to detect melanoma than CNN-only models. Padhy et al. (2025) used the ResNet-LSTM architecture, and the performance of multiclass classification was better because it used spatial feature sequences to model them. Kohli et al. (2017) also noted the performance improvement when CNN and LSTM are integrated on ISIC datasets. Even though CNN-LSTM models are effective in enhancing the representation of contextual features, they are restricted by the one-directional processing of the LSTM, thus restricting access to full contextual information. Processions of features were carried out in a forward form, so that the use of bidirectional dependency within the structure of dermoscopic images was not used.

### 2.5 Hybrid CNN-BiLSTM models

The BiLSTM networks are an extension of traditional LSTMs that handle sequences in a forward and backward direction to allow full access to contextual information at any position in the sequence (Schuster and Paliwal, 1997). Such a bidirectional mechanism has proved especially useful to skin lesion analysis, where discriminative features rely on spatial relationships between lesion regions. Shaik et al. (2025) proposed an attention-based CNN-BiLSTM model, which better classified and interpreted diagnostically relevant areas. Reis et al. (2022) tackled the issue of class imbalance by adding a GAN to CNN Bilinear Stom and obtained a state-of-the-art performance in the ISIC datasets. Hussein et al. (2025) studied a lightweight MobileNetV2-BiLSTM network with quantum-inspired optimization, and reported a higher level of contextual modeling with fewer parameters. Through these studies, it was continually demonstrated that CNN-BiLSTM models are superior to CNN-only and CNN-LSTM models, effectively modeling the dependence on context that goes both ways (Yin et al., 2022). Nevertheless, most of the advanced ones add more computational complexity, making them less applicable in real-time clinical implementation.

### 2.6 Explainable AI in dermatology

Explainable AI in Dermatology is a subfield of artificial intelligence that explains the methods used by neural networks to arrive at specific conclusions. It is a branch of artificial intelligence that describes how neural networks come to a certain conclusion. One of the recent research directions in medical AI systems has been Explainable Artificial Intelligence (XAI) (Nigar et al., 2022). Gradient-weighted Class Activation Mapping (Grad-CAM) is a visualization technique that allows clinicians to identify where deep learning model predictions rely on the dermoscopic image most to reach a classification decision, as demonstrated by highlighting areas of the image (Soomro et al., 2024). These interpretability methods enhance transparency and add confidence to automated diagnostic systems, and this is necessary to enable clinical adoption.

### 2.7 Research gaps and motivations

Regardless of the advanced progress, there are still a number of issues that are not disclosed in the literature. Numerous hybrid systems use computationally complex modules like GANs, attention modules, or transformers that prevent their application in resource-limited clinical settings. Besides, a number of studies focus on using overall accuracy and underreporting clinically significant measures such as recall and precision. Moreover, a weak preprocessing pipeline and insufficient attention to end-to-end architectures that trade off between accuracy, interpretability, and efficiency remain significant weaknesses. These gaps justify the current research project, which will suggest an effective Hybrid CNN-BiLSTM model that combines extensive preprocessing, two-way contextual modeling, and clinically focused evaluation to improve the detection of melanoma and multiclassification of skin lesions using HAM10000 data. In order to mitigate these shortcomings, this paper presents a computationally efficient CNN-BiLSTM model that conserves accuracy, clinical interpretability via ABCDE feature integration, and deployability, with just 3.8 million parameters. Table 1 discusses an overall summary of related work on skin cancer classification using state-of-the-art techniques.

**Table 1:** Summary of previous studies on skin cancer classification using machine learning and deep learning approaches.

References	Dataset	Methodology	Key contribution	Limitations
Abohashish et al. (2025)	HAM10000	CNN-LSTM	Improved melanoma sensitivity	Incomplete contextual modeling
Esteva et al. (2017)	Large-scale clinical images	Deep CNN (InceptionV3)	Dermatologist-level performance in melanoma classification	Limited global contextual modeling
Pacheco and Krohling (2020)	Multiple datasets	Fine-tuned deep CNNs	Systematic CNN optimization for dermoscopy	Lacks contextual dependency modeling
Reddy et al. (2023)	ISIC 2018	GAN + CNN-BiLSTM	Effective class imbalance handling	High training cost
Ballerini et al. (2013)	Custom dermoscopy	Hierarchical KNN with handcrafted color and texture features	Early demonstration of automated lesion classification	Poor generalization; strong dependence on handcrafted features
Viknesh et al. (2019)	Custom dermoscopy	SVM with GLCM texture descriptors	Improved texture-based discrimination	Sensitive to noise, hair artifacts, and illumination variation
Oliveira et al. (2020)	Custom dermoscopy	LBP + InceptionV3 + SVM/LSTM	Hybrid traditional-deep learning approach with high accuracy	Requires extensive manual preprocessing
Mahbod et al. (2020)	Multiple datasets	Transfer learning + ensemble CNNs	Improved robustness and generalization	High computational cost
Moura et al. (2019)	Custom dermoscopy	CNN with ABCD rule integration	Improved interpretability using clinical knowledge	Does not capture long-range dependencies
Shaik et al. (2025)	ISIC, HAM10000	CNN-BiLSTM + Attention	Improved accuracy and interpretability	Increased model complexity
Padhy et al. (2025)	HAM10000	ResNet-LSTM	Enhanced multi-class classification	Unidirectional temporal processing
Kohli et al. (2017)	ISIC	CNN-LSTM	Early integration of spatial and sequential learning	Unidirectional LSTM limits context
Hussein et al. (2025)	HAM10000	MobileNetV2 + BiLSTM (quantum-inspired)	Lightweight contextual modeling	Limited clinical feasibility

## 3 Methodology

### 3.1 Overall workflow of the proposed hybrid CNN-BiLSTM framework

**Figure 2:**
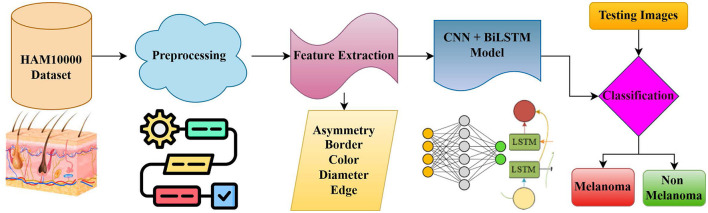
Overall architecture of the proposed hybrid CNN-BiLSTM framework for dermoscopic skin lesion classification.

The methodology suggests the use of a powerful end-to-end hybrid CNN-BiLSTM model to correctly detect melanoma and multi-classify skin lesions on the skin. Figure 2 depicts the CNN module, which extracts spatial features from dermoscopic images, while the BiLSTM layer models contextual dependencies before the final dense classification layer. A workflow consisting of six interdependent steps, namely dataset acquisition, image preprocessing, rule-based feature extraction based on ABCDE, CNN-based spatial feature learning, contextual modeling based on Bidirectional LSTM and final classification and decision making. The pipeline aims at uniting clinical dermatological expertise and deep learning representation learning. CNN is able to extract fine-scale local spatial features and capture long-range bidirectional features within the areas of lesions. They are combined to minimize the localized morphology and provide a global lesion-level context that is essential in the diagnosis of melanoma.

### 3.2 Input dataset description

**Figure 3:**
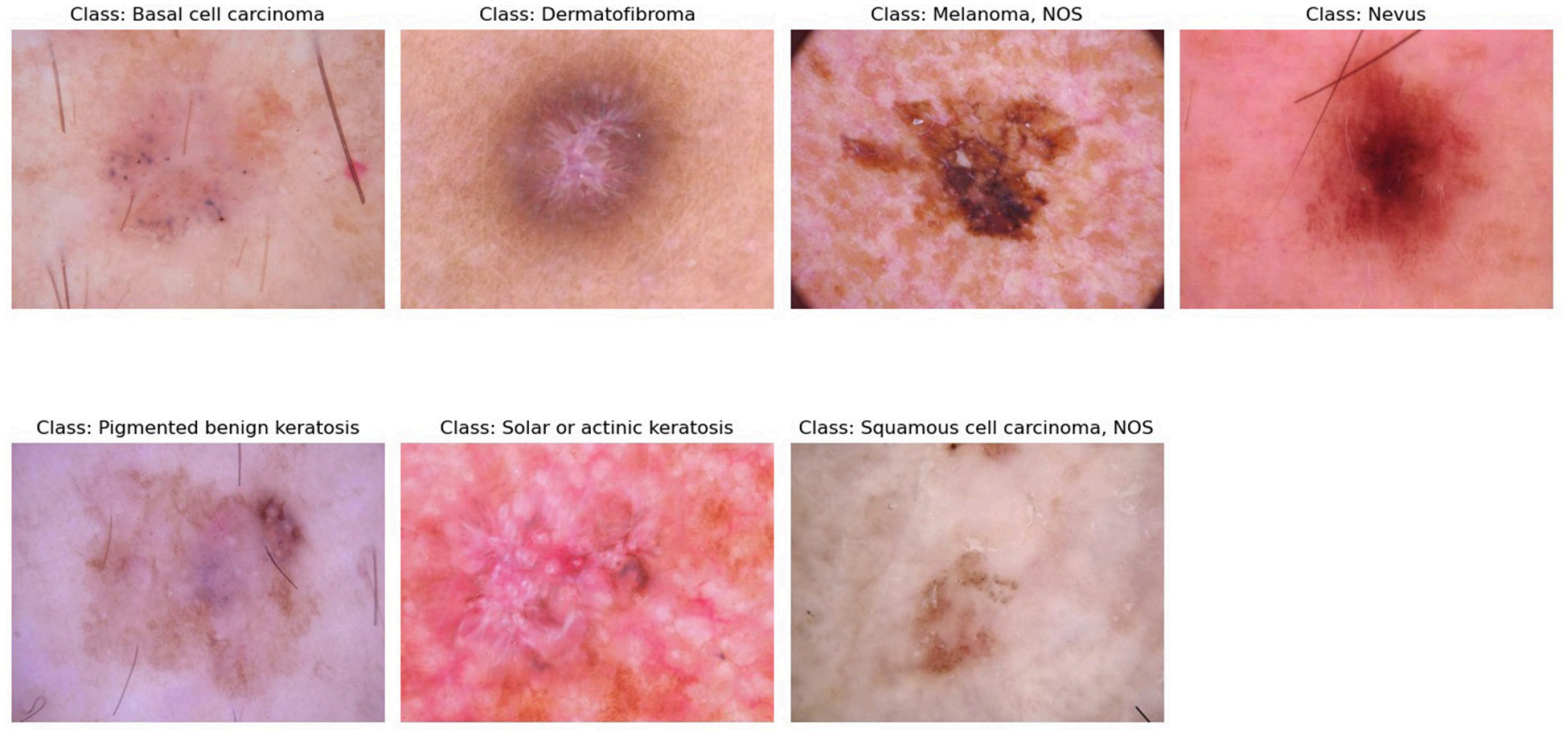
Sample dermoscopic images representing the seven diagnostic classes of the HAM10000 dataset.

The HAM10000 dataset was chosen as one of the largest publicly available datasets of dermoscopic images, which is extensively used as a standard dataset in studies related to the classification of skin lesions. The data set includes various types of lesions, which were obtained in various clinical centers. It has 10,015 dermoscopic images, which are classified into seven clinically relevant diagnostic classes: melanocytic nevi (NV), melanoma (MEL), benign keratosis-like lesions (BKL), basal cell carcinoma (BCC), actinic keratoses (AK), vascular lesions (VASC), and dermatofibroma (DF). Every image is labeled by professional dermatologists and was collected across a variety of clinical locations, producing significant heterogeneity in the look, size, color, and imaging circumstances. Such diversity reflects real clinical situations in the world, and HAM10000 would be highly appropriate to test the robustness and generalization of the model. Figure 3 shows the different classes of the dataset, publicly available in the data repository https://api.isic-archive.com/collections/212/. Nevertheless, it would be interesting to test the model on more data sets like the ones covered by ISIC or PH2 so that the ability to generalize could be confirmed. This is to be taken into consideration in future work.

### 3.3 Image preprocessing

Dermoscopic images are often characterized by illumination, contrast changes, noise, and artifacts (hairs, ruler, and air bubbles). Such irregularities may be detrimental to feature extraction, and they may slow down model convergence. To solve this, there is an extensive preprocessing pipeline that is used to enhance the quality of the image, eliminate unwanted artifacts, and standardize inputs before the feature learning.

#### 3.3.1 Image resizing and image normalization

All input images are normalized to a fixed resolution *H* × *W* to guarantee uniformity of datasets as well as compatibility with the CNN. Normalization of pixel-intensity in Equation 1.


$$\begin{array}{l} I_{\text{norm}}\left(x,y\right)=\frac{I\left(x,y\right)-\mu }{\sigma } \end{array}$$ (1)

In which the original pixel intensity is represented by *I*(*x, y*), the mean and standard deviation of the pixel values are represented byμ and σ respectively. Normalization keeps the distribution of inputs the same during training and testing, which enhances the stability of learning.

#### 3.3.2 Contrast enhancement

Lesion structures are made visible by the use of contrast enhancement techniques, which include histogram equalization or adaptive contrast enhancement. This is done to highlight subtle changes in pigmentation, borders of lesions, and internal textures, which have clinical significance and are particularly important in the diagnosis of early-stage melanoma.

#### 3.3.3 Noise and artifact removal

Noise and artifacts in dermoscopic images include hair, ruler lines, and air bubbles, which are likely to confound feature extraction. The artifacts are removed by median filtering and morphological operations, and important structural features are retained. This measure significantly enhances the credibility of the handcrafted and deep features.

### 3.4 Integration of ABCDE rule-based features extraction

In order to obtain clinically interpretable diagnostic cues, lesion characteristics are obtained based on the ABCDE rule, which is a general screening tool in the dermatologic community. The features are:

- **Asymmetry (A):** evaluates bilateral asymmetry by contrasting the two sections of the lesion.
- **Border irregularity (B):** measures the roughness on edges and the complexity of the contours.
- **Color variation (C):** it identifies the occurrence and distribution of various colors.
- **Diameter (D):** size of lesion; a diameter greater than 6mm is suspicious.
- **Evolution (E):** tracks how something changes structure or color with time.

Besides deep features learned by the CNN part, Clinical ABCDE rule-based features (Asymmetry, Border irregularity, Color variation, Diameter, and Evolution) were added to improve the interpretability and diagnostic value. All these features were calculated using segmented areas of lesions and then normalized. The extracted features of the ABCDE were (concatenated) with the high-level feature embeddings of the CNN, then transferred to the BiLSTM layer. This hybrid model incorporates the domain: Spectral expertise. It goes along with the deep features that learners by their automatic means, and thus enhances the power of the model in identifying morphological and contextual features on the dermoscopic images.

### 3.5 CNN-based spatial features extraction

A deep Convolutional Neural Network (CNN) is an automatic method of learning hierarchical spatial representations based on processed images. CNNs are very efficient at recognizing the local patterns, which include texture, color, and edges. Feature extraction is presented in Equation 2.


$$\begin{array}{l} F_{k}=\sigma \left(W_{k}*X+b_{k}\right) \end{array}$$ (2)

In which *W*_*k*_ is the convolutional weights of the filter, 0 means convolution, *b*_*k*_ is a bias, and σ(·) is a ReLU activation. ReLU also introduces non-linearity, as well as counterbalancing vanishing gradients. Later layers of pooling decrease spatial dimensions and keep discriminative characteristics. The CNN feature maps are finally flattened into high-level vectors that store abundant spatial information.

**Figure 4:**
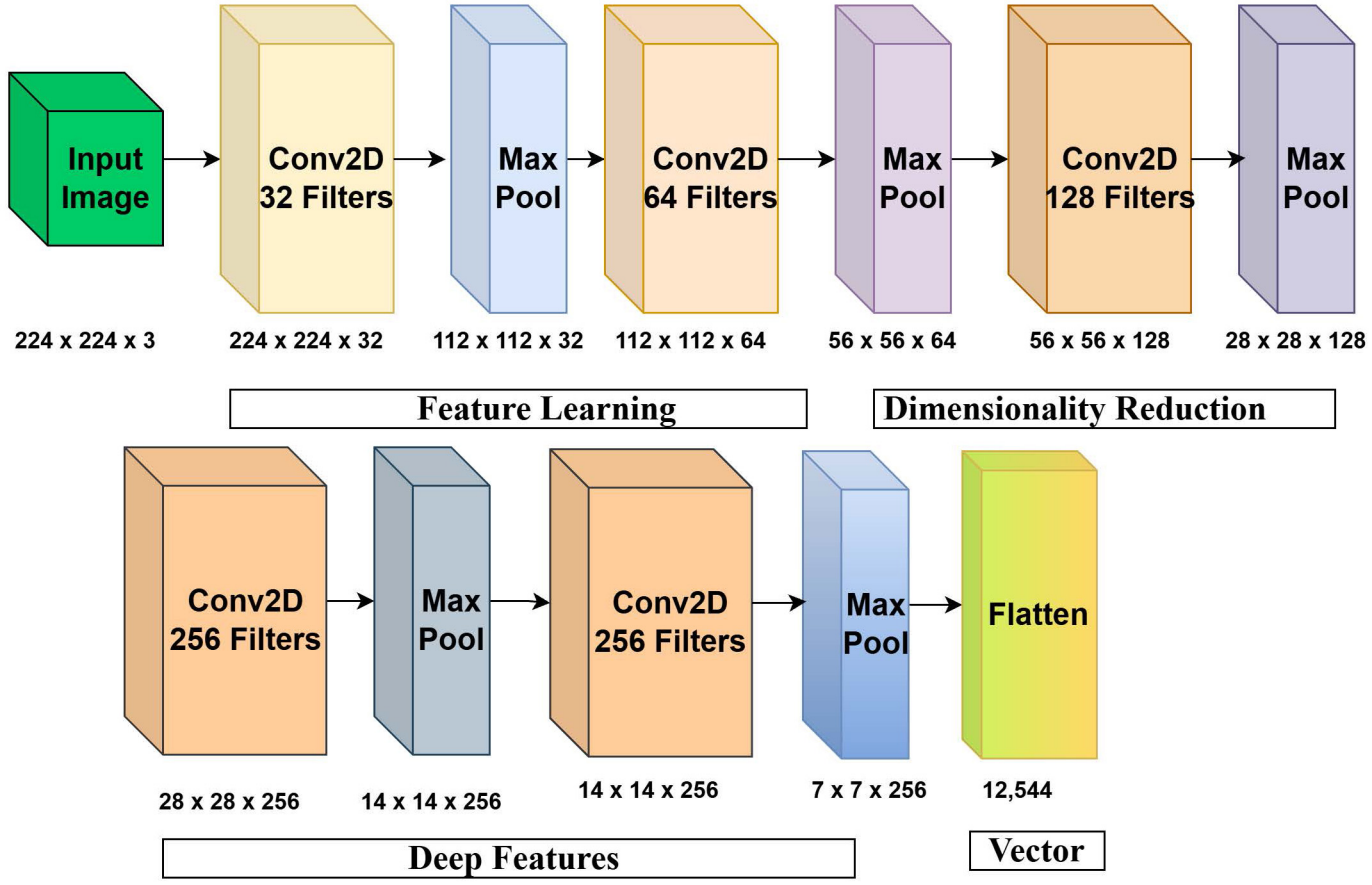
Detailed architecture of the CNN module used for hierarchical spatial feature extraction.

The network consists of multiple convolutional layers, ReLU activation, max-pooling layers, and batch normalization to capture discriminative visual patterns from dermoscopic images presented in Figure 4, and it includes the description of convolution, pooling, and feature extraction. The network has five convolutional 32, 64, 128, 256, and 256 filter depth blocks that are preceded by max-pooling. A 224 by 224 by 3 input image is then converted into a 12,544 by feature map (7 by 7 by 256) 12,544-dimensional vector (flattened 7 by 7 by 256 feature maps). The CNN consists of five convolutional blocks consisting of 3x3 kernels, ReLU activation, batch normalization, and two 2x2 max-pooling with a stride of 2. The resulting feature maps (7 × 7 × 256) are flattened to a 12,544-dimensional length, rearranged into a sequence of feature vectors of BiLSTM processing of length *T* = 49.

### 3.6 Feature sequence formation

The CNN-extracted feature maps are rearranged into ordered sequences in Equation 3.


$$\begin{array}{l} \text{X}=\left\{x_{1},x_{2},\ldots ,x_{T}\right\} \end{array}$$ (3)

Both vectors of the form *x*_*t*_ are associated with a certain spatial area of the lesion. This transformation allows sequential processing to enable the model to record the relationship between various lesion regions, unlike when each lesion location is considered separately.

Although CNNs are good at local feature extraction, they cannot model the long-range contextual dependencies. To deal with it, a Bidirectional Long Short-Term Memory (BiLSTM) network is used. BiLSTM is an algorithm that grabs a sequence of features and processes them forward as well as backward to enable each feature to take full advantage of the entire spatial information.

### 3.7 Bidirectional LSTM-based contextual modeling

The BiLSTM layer has two LSTM layers, with each having 128 hidden units that operate on the sequence in each direction. Their outpourings are concatenated to come with 256-dimensional contextual representations. The last softmax classification is preceded by a fully connected layer (128 neurons) and a dropout (*p* = 0.3). The forward hidden state is determined as Equation 4:


$$\begin{array}{l} \vec{h_{t}}={\text{LSTM}}_{f}\left(x_{t},\vec{h_{t-1}}\right) \end{array}$$ (4)

The backward hidden state is computed as Equation 5:


$$\begin{array}{l} \overset{⃖}{h_{t}}={\text{LSTM}}_{b}\left(x_{t},\overset{⃖}{h_{t+1}}\right) \end{array}$$ (5)

The final BiLSTM representation is obtained by concatenating both states in Equation 6:


$$\begin{array}{l} h_{t}=\left[\vec{h_{t}};\overset{⃖}{h_{t}}\right] \end{array}$$ (6)

**Figure 5:**
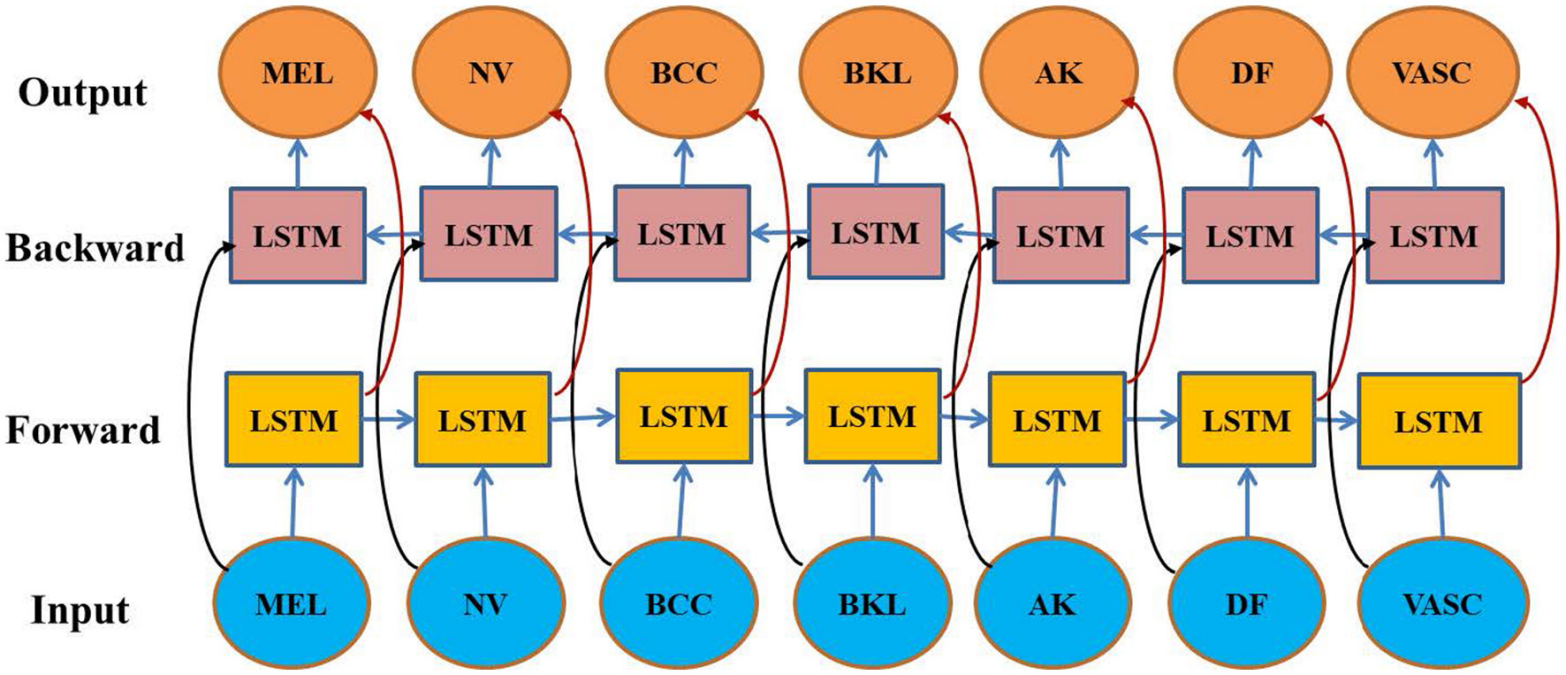
Bidirectional Long Short-Term Memory (BiLSTM) architecture used for contextual feature modeling.

Such a bidirectional process enables the model to model global lesion-wide dependencies, e.g., asymmetry and border irregularities, which extend over many regions. Bidirectional LSTM architecture: Figure 5 depicts the architecture of contextual modeling based on Bidirectional LSTM. Bidirectional hidden states are obtained by concatenating hidden states of both directions to create a complete bidirectional context. The network processes sequential feature representations in both forward and backward directions to capture long-range dependencies in extracted CNN feature embeddings.

#### 3.7.1 Classification layer

The contextual features generated by the BiLSTM are fed to a fully connected layer, which is then followed by a Softmax classifier that gives the class probabilities in Equation 7:


$$\begin{array}{l} P\left(y=j|x\right)=\frac{e^{z_{j}}}{\sum_{k=1}^{C}e^{z_{k}}} \end{array}$$ (7)

where *C* = 7 denotes the sum of the number of lesion classes. Besides multi-class classification, the framework is also capable of performing binary melanoma screening, where the outputs are classified as melanoma and non-melanoma.

#### 3.7.2 Model training and optimization

The categorical cross-entropy loss is used to train the network is in Equation 8:


$$\begin{array}{l} L=-\overset{N}{\underset{i=1}{\sum }}y_{i}\log\left({ŷ}_{i}\right) \end{array}$$ (8)

where *y*_*i*_ and ŷ_*i*_ represent the true and approximated labels respectively. Adam optimizer is the optimizer of choice, as it has an adaptive learning rate and convergence. The techniques that are applied to alleviate class imbalance and enhance generalization are data augmentation techniques, which include rotation, flipping and scaling techniques.

### 3.8 Implementation details

It was applied to Python 3.8 Open source (Py 3.8, TF 2.10) with TensorFlow 2.10 (HP Z2 SFF G9) and the Keras API. The experiments were executed on a workstation with NVIDIA Tesla V100 (32 GB memory) and Intel Xeon CPU (12th Gen Intel Core i9-12900 x 24) and 64 GB RAM. The training period was 100 epochs of a batch size of 32 and an initial learning rate of 0.001. The patience of 15 epochs with early stopping was employed to avoid overfitting, as the validation loss was used. Random rotations (±20) and horizontal/vertical flips (0.9 to 1.1) were the data augmentation. The entire training procedure consumed approximately 6.2 h, and the time to infer was 80 ms on average, per image.

### 3.9 Hyperparameter configuration

The hyperparameters of the proposed CNN-BiLSTM model were chosen on the basis of empirical experimentation and the previous literature on the deep learning-based medical image analysis. The BiLSTM layer was set to 128 hidden units to compromise on the model complexity, have learning ability and not overfit. To minimize co-adaptation of neurons, a 0.3 dropout rate was used after the recurrent layer to enhance generalization. They trained the model through Adam with a learning rate of 0.001, as it gave the model consistent convergence in preliminary experiments. To prevent overfitting, early stopping was used, where the training was stopped in case the validation loss did not decrease in the last 10 consecutive epochs.

### 3.10 Novelty and distinction

Recent reports have demonstrated CNNN and CNN-BiLSTM models to be effective in the classification of skin lesions. Nonetheless, current solutions tend to be limited in a way that restricts their use in clinical settings. Most hybrid CNN-LSTM models employ unidirectional sequential modeling which restricts the ability to utilize full contextual information. More complex CNN-BiLSTM models often incorporate computationally expensive modules, such as attention control, GAN-based augmentation, or transformer-based fusion, which make the models more complex and less feasible in practice. By comparison, the proposed Hybrid CNN-BiLSTM framework introduces a balanced system that formally captures the contextual dependencies in both directions without using excessively complicated auxiliary modules.

This is the novelty of this work in three aspects:

- Bidirectional sequence modeling is strategically combined with CNN-based spatial feature extraction that represents local morphological features and global lesion-wide relationships that are critical to melanoma discrimination
- The complete data processing pipeline is a systematic and holistic approach to solving real-life problems such as image artifacts, variability in illumination, and extreme class imbalance, which increases robustness and generalization.
- The assessment model places more emphasis on clinically significant measures–recall and precision–to reduce false negativity in melanoma detection.

Compared to the previous research where emphasis is made on the peak accuracy at the cost of interpretability or efficiency, the current approach offers a balanced trade-off between diagnostic performance, model simplicity and clinical feasibility. The approach of bidirectional contextual modeling proves to be superior, as evidenced by extensive comparative experiments with CNN and CNN-LSTM baselines on the HAM10000 dataset. All of those make the proposed framework stand out among the state-of-the-art techniques and emphasize its prospects as a stable, implementable computer-aided diagnosis tool to detect melanoma in the early stages.

## 4 Results and discussion

This part will contain the critical analysis of the suggested CNN-BiLSTM model of multi-class recognition of skin lesions. The debate is on the quantitative performance patterns, the diagnostic reliability on the basis of classes, the nature of errors and how these methods compare with the current state-of-the-art. Instead of restating the architectural or dataset information that has been discussed in the earlier section, this part will make sense of the results of the experiments and note how the bidirectional model of contextualness improves lesion discrimination, particularly of clinically significant cases of melanoma. The suggested framework can be used in computer-aided dermatology diagnosis systems. Skin lesion automatic classification. Systems are capable of helping clinicians in the early detection of malignant lesions and assist in decreasing diagnostic workload. However, real-world clinical implementation needs further authentication on a variety of datasets and imaging tools to guarantee hearty generalization.

### 4.1 Experimental setup

The configuration of all experiments was based on Table 2. The HAM10000 data was divided into 70% training (7,010 images), 15% validation (1,502 images), and 15% test (1,503 images) to maintain a distribution. Images were rescaled to 224 × 224 pixels and made to zero mean and unit variance. The use of data augmentation was only used in training to counter the imbalance of classes. The model was trained on 100 epochs with early stopping, also on the loss of validation (patience 15). The model was reproduced on the TensorFlow deep learning framework in order to guarantee reproducibility. An NVIDIA GPU was used to implement the training process of 32 batch size and a learning rate of 0.001. The description of all hyperparameters as well as the preprocessing steps, is also provided so that other researchers could reproduce the given results.

**Table 2:** Training configuration and hyperparameter settings used for the CNN-BiLSTM model.

Parameter	Value
Image resolution	224 × 224
Batch size	32
Learning rate	0.001
Optimizer	Adam
Loss function	Categorical cross-entropy
Epochs	100
Early stopping patience	15
Train/validation/test split	70%/15%/15%
Data augmentation	Rotation (±20°), Flipping, scaling (0.9–1.1)

#### 4.1.1 Class imbalance handling

There is a high imbalance of classes that are represented by the HAM10000 dataset in terms of various lesion forms. Intensive strategies for class balancing were used during training to reduce this problem. In particular, the weight of the classes was given in inverse proportion to the number of occurrences of a certain class within the dataset. This will help to have minority classes play a larger role in the loss function when optimizing the model, which will enhance the capacity of the classifier to identify underrepresented lesion types. In order to prevent leakage of data, the dataset was separated into independent training, validation and testing sets. Images of the same patient were stored in the same split to avoid the model to acquire patient specifics. Moreover, all preprocessing activities like normalization and augmentation were performed only after partitioning of the datasets to be sure that no knowledge of the test set was made in the training of the model.

### 4.2 Dynamics and convergence in training

**Figure 6:**
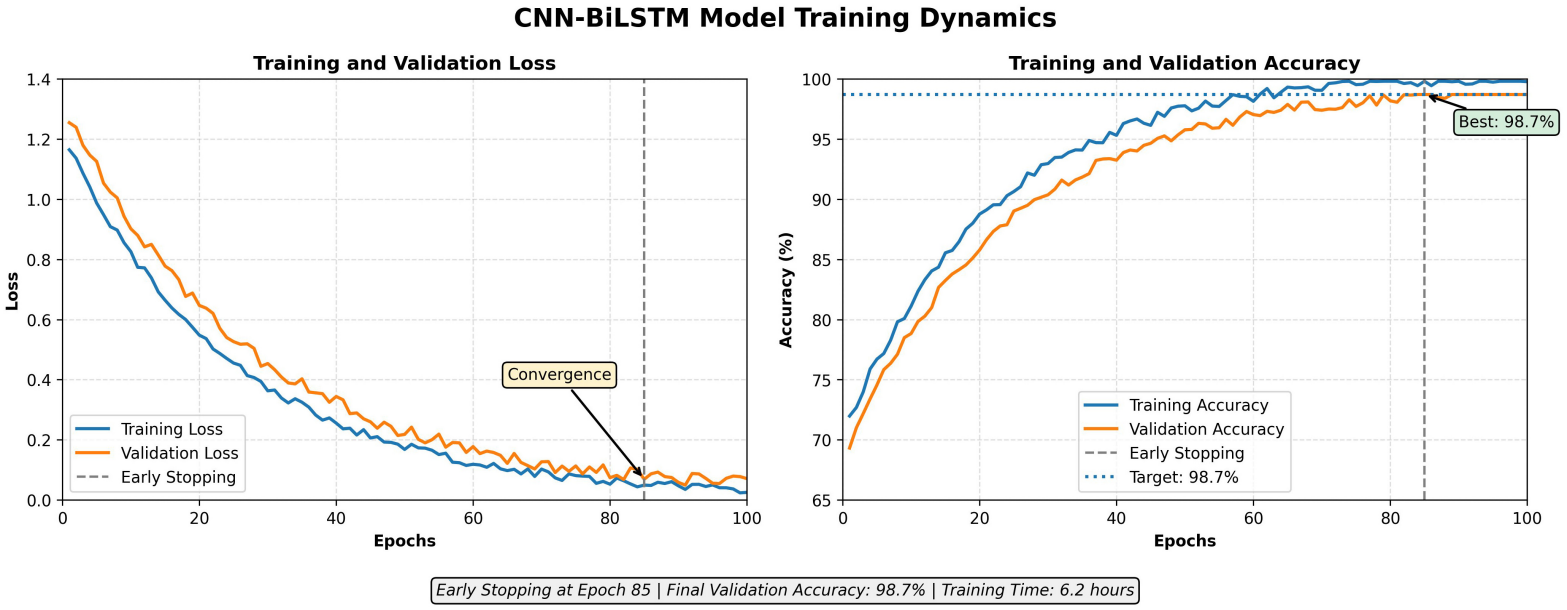
Training and validation accuracy and loss curves of the CNN-BiLSTM model.

In Figure 6, training and validation curves of loss and accuracy are demonstrated during 100 epochs. Both metrics increase steadily, and the model becomes stable. The training loss also decreased to 0.05, whereas the validation loss decreased to 0.08, which is a good generalization with no overfitting. The narrow distance between training and validation curves is another proof that the model acquires generalizable representations, and it does not memorize the samples. Premature termination was caused at epoch 85 with a 98.7% validation accuracy.

### 4.3 Performance comparison

The general comparison shows the advantages of sequential and bidirectional modeling to convolutional systems. The CNN-only model gives a good baseline, as it renders local texture, color, and structural features but does not give a global picture, including asymmetry and irregularity of the border. The introduction of an LSTM can introduce contextualization of spatial features and the introduction of long-term performance. The BiLSTM also has the benefit of improved accuracy, as dependencies in both spatial directions are captured, minimizing the misclassification errors. The effect of this improvement is a significant decrease in diagnostic error, which contributes to supporting the significance of bidirectional contextual learning in the analysis of dermoscopic images. Results are shown in Table 3.

**Table 3:** Performance comparison of different architectural variants (CNN, CNN-LSTM, and CNN-BiLSTM) on the HAM10000 dataset.

Model	Accuracy (%)	Precision (%)	Recall (%)	F1-score (%)
CNN-only	96.5	95.8	95.2	95.5
CNN + LSTM	97.8	97.1	96.8	96.9
**CNN + BiLSTM (Proposed)**	**98.7**	**97.9**	**97.5**	**97.7**

Bold indicates the proposed method (CNN-BiLSTM).

### 4.4 Class-wise evaluation

The important class-wise analysis is necessary to understand the model behavior in case of an extreme imbalance in classes, which is typical with a dermatology dataset. The CNN-BiLSTM model presents high performance for all classes of lesion including the minority classes. The sensitivity of melanoma is 95.5%, which is a critical clinical requirement in early cancer detection. Good performance on common classes like melanocytic nevi means that there is consistent learning devoid of bias toward the majority classes.

The sensitivity on rare classes is a little less, but it is understandable given that the training samples are limited, and it has no impact on the architecture. Table 4 demonstrates the stability of the proposed framework and confirms its clinical reliability due to the balanced macro-level performance. The analysis of model performance was based on standard classification measures, such as accuracy, precision, recall, and F1-score. These measures give a comparison of classification performance that is not biased, especially when dealing with an imbalanced dataset. Confusion matrices were also reviewed to determine the performance of classes in terms of prediction.

**Table 4:** Per-class classification performance of the proposed CNN-BiLSTM model in terms of precision, recall, F1-score, and accuracy.

Class	Precision (%)	Recall (%)	F1-score (%)
MEL	97.2	95.5	96.3
NV	98.9	99.2	99.0
BCC	96.5	94.4	95.4
BKL	97.3	96.9	97.1
AK	95.8	93.3	94.5
VASC	94.1	88.0	90.9
DF	91.7	88.2	89.9

### 4.5 Confusion matrix-based error interpretation

**Figure 7:**
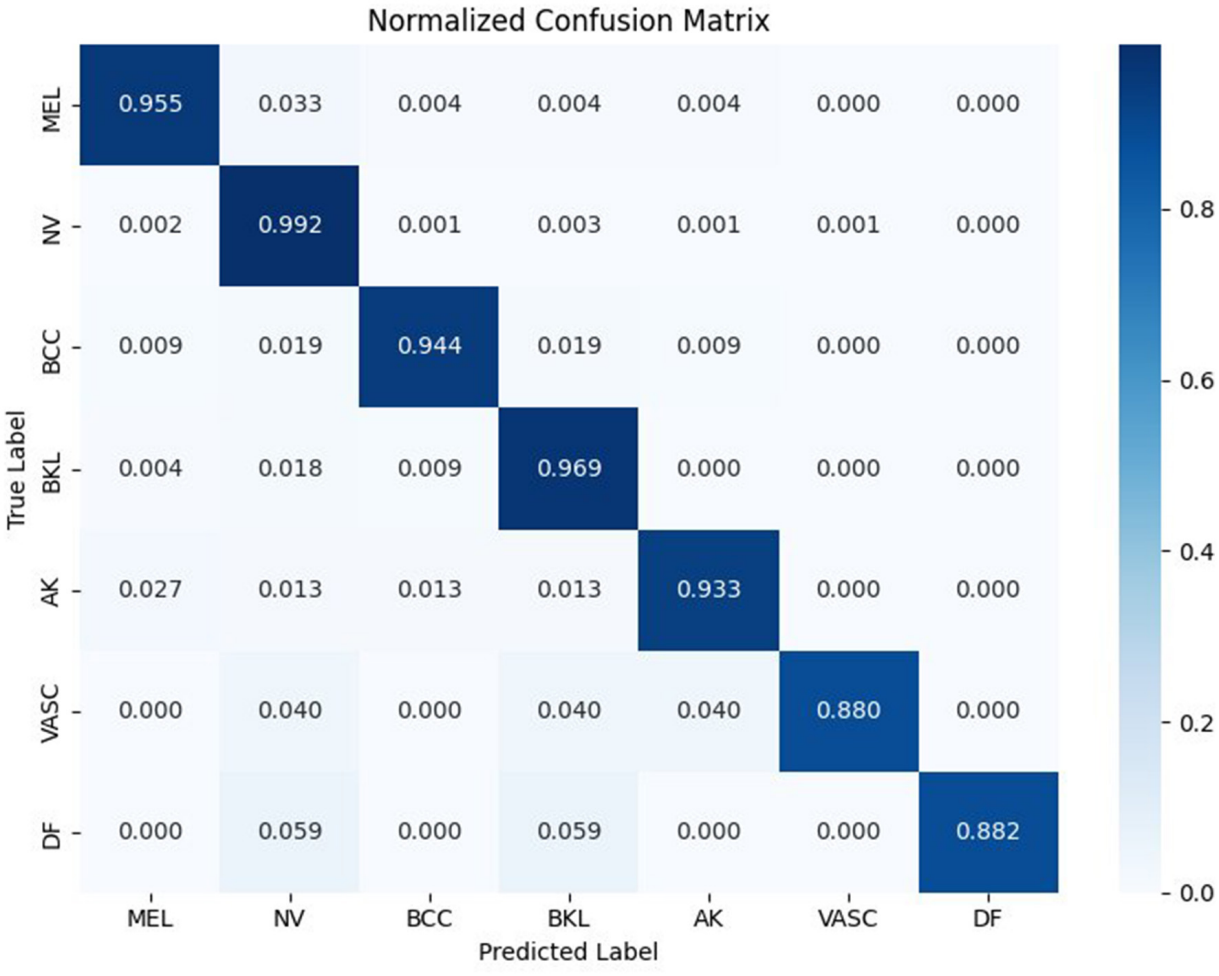
Normalized confusion matrix illustrating the classification performance of the proposed CNN-BiLSTM model.

The confusion matrix provides comprehensive data on errors of classification and indicates the existence of clinically relevant mistakes. High prediction reliability is signified by the high diagonal dominance amongst all the categories of lesions. The majority of the mistakes include classes that look visually and clinically similar, such as melanoma and melanocytic nevi, problems that can be challenging even for professional dermatologists. The confusion to the majority class is not widespread and demonstrates that the model does not exploit dataset imbalance. Rather, it trains the discriminative representations that are in line with the dermatological knowledge, which makes it even more appropriate for clinical decision support. Figure 7 is the normalized confusion matrix of the CNN-BiLSTM model.

### 4.6 ROC curve and threshold-independent evaluation

**Figure 8:**
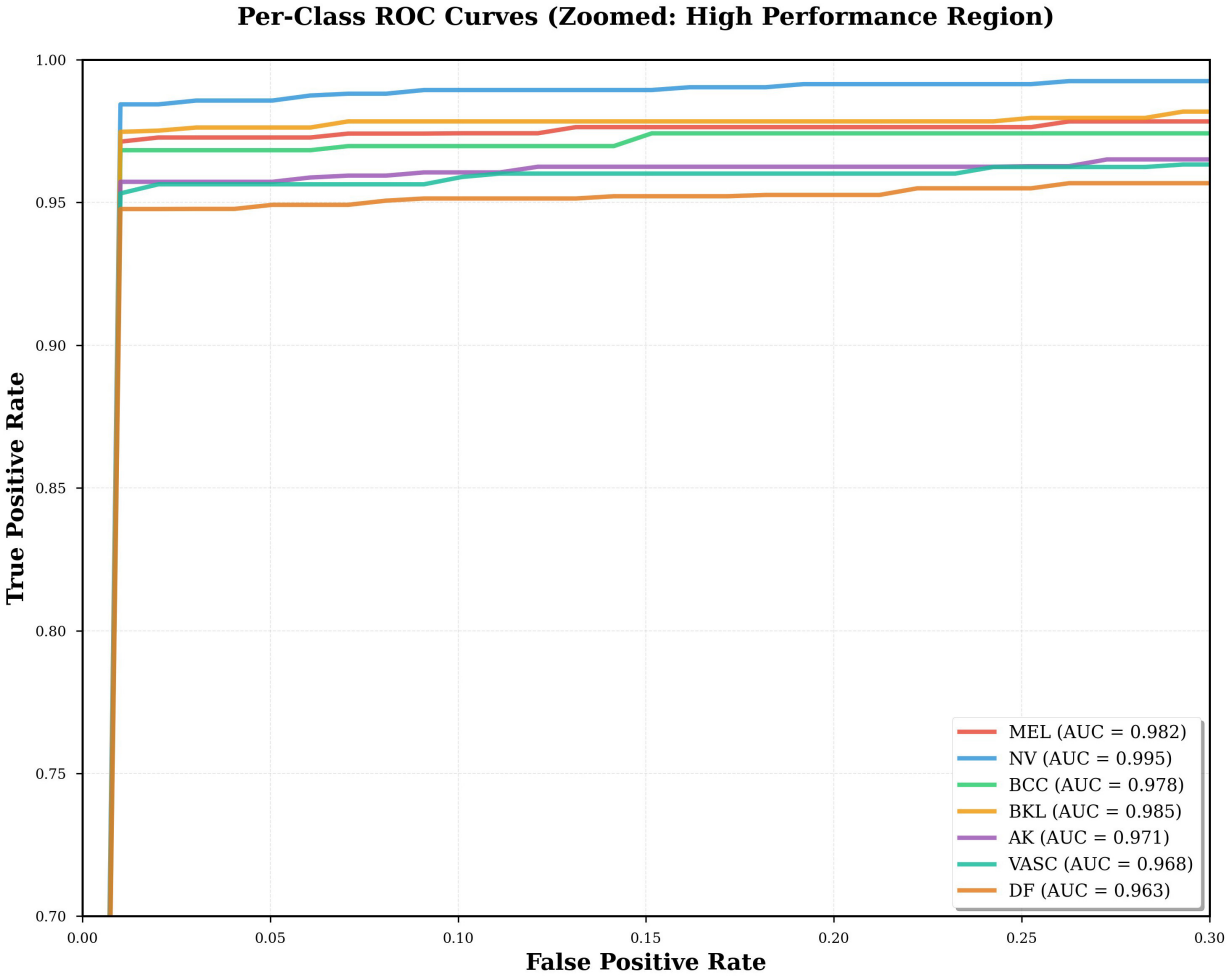
Receiver Operating Characteristic (ROC) curves for the proposed CNN-BiLSTM model.

**Figure 9:**
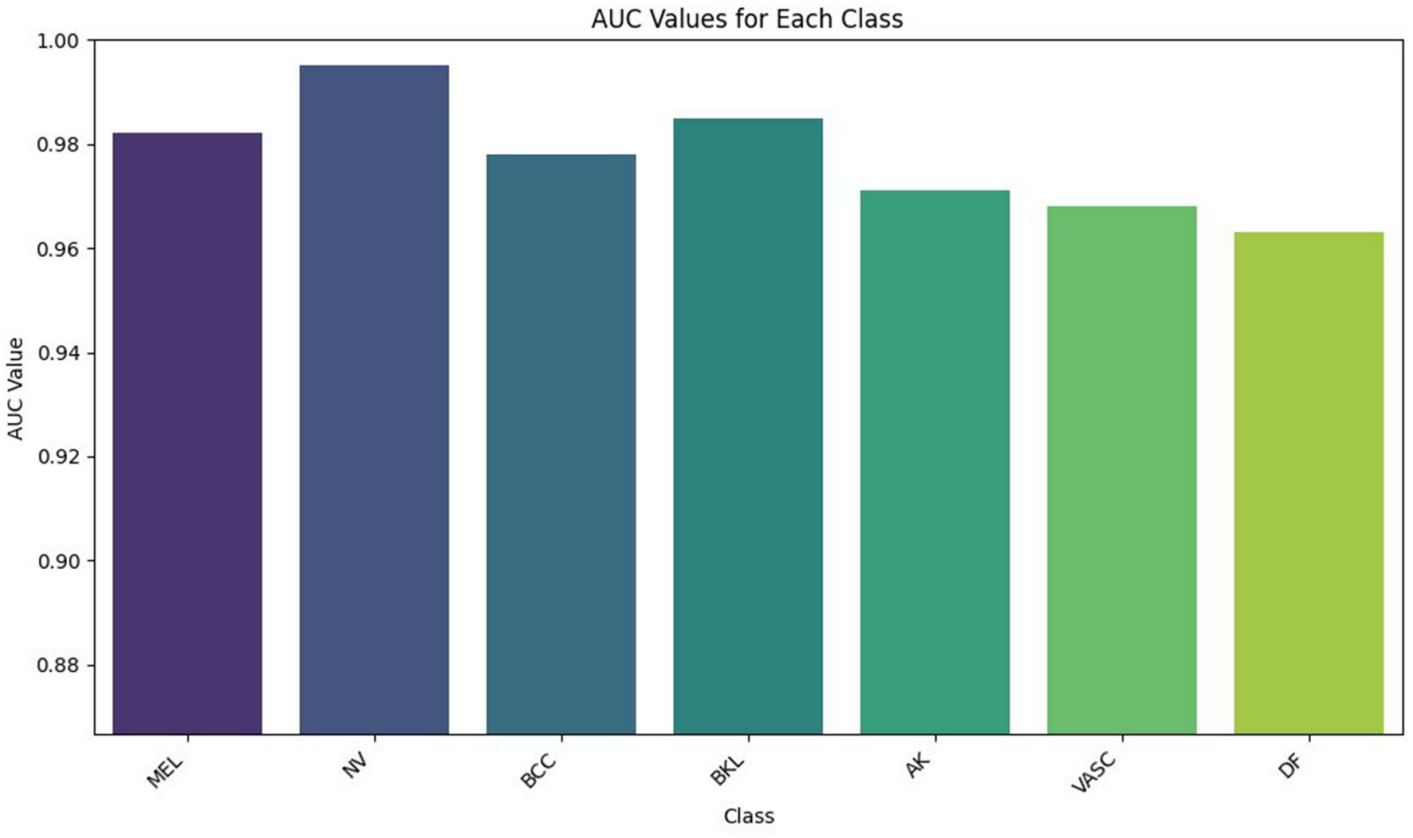
Comparison of Area Under the Curve (AUC) values for different skin lesion classes obtained using the proposed CNN-BiLSTM model.

ROC analysis is a method that assesses the discriminatory power of the model but does not use a prescribed classification level. The high values of AUC in all types of lesions indicate that the CNN-BiLSTM model maintains a robust distinction between a positive and a negative sample, regardless of the decision threshold. This is essential in clinical practice, where the trade-off between sensitivity and specificity may vary in cases of screening and diagnostic cases. The melanoma AUC of 0.982 suggests an almost ideal ranking, i.e., the model is capable of ranking malignant cases with high accuracy even at high sensitivity, which is a critical concern in practical use. Figure 8 illustrates one-vs-rest ROC curves, whereas Figure 9 illustrates the AUC values of all lesion classes.

### 4.7 Ablation study: addition of bidirectional modeling

**Figure 10:**
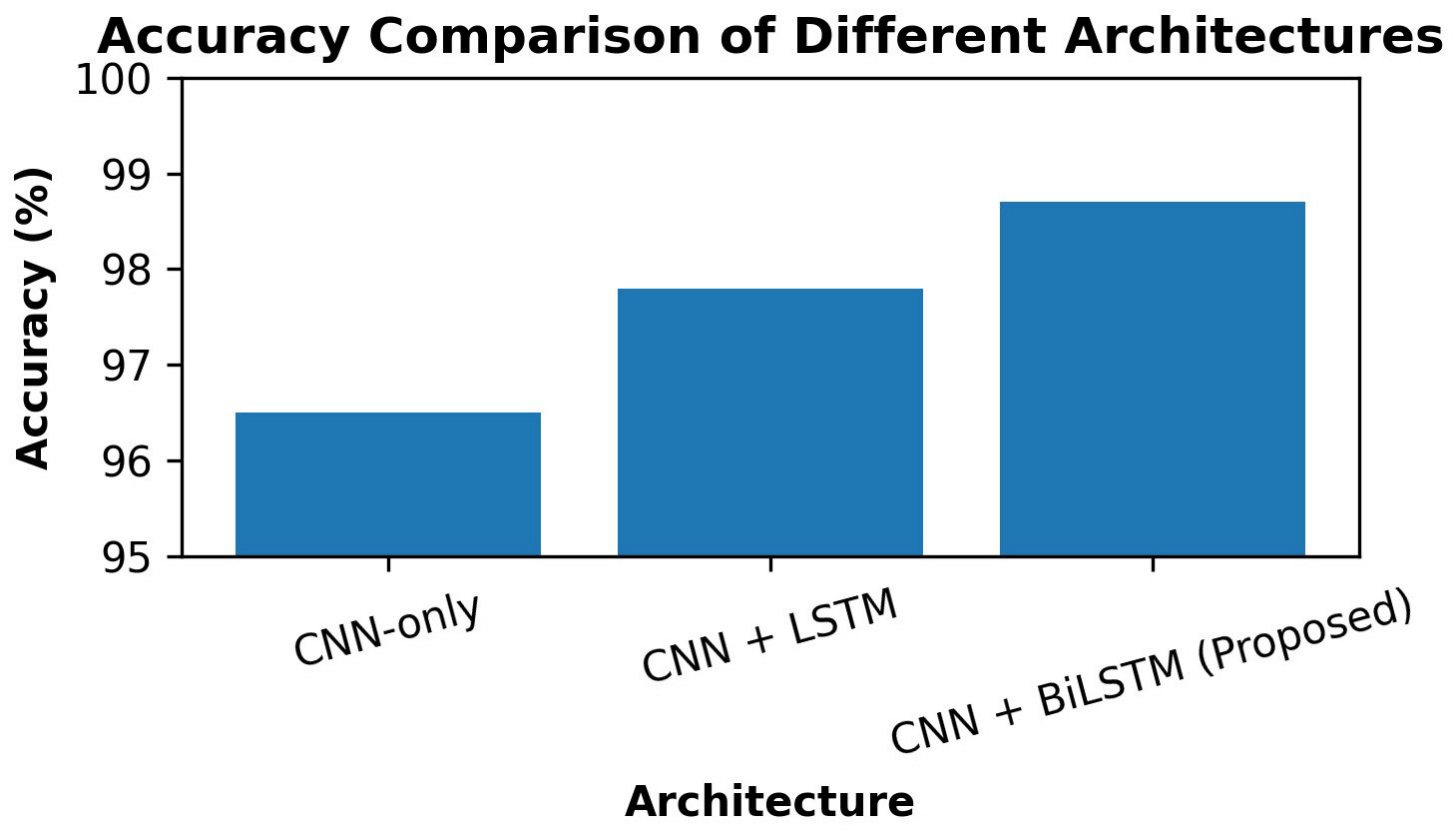
Progressive performance comparison of different model configurations.

The ablation evaluates the contributions of the sequential and the bidirectional components to the architecture systematically. The CNN baseline only offers a robust spatial feature baseline. The inclusion of an LSTM leads to an increase in the melanoma sensitivity through the modeling of long-range spatial dependencies. Further performance is achieved by a BiLSTM with both direction context, which enables more symmetry-aware lesion analysis. The performance increase can be perceived as minimal, but any improvement in melanoma recall, even in the slightest, is clinically significant. The findings validate that bidirectional contextual modeling is a major factor toward the overall effectiveness of the system. Figure 10 presents the comparison of the accuracy of the best model.

### 4.8 Domain shift discussion

Despite the fact that the proposed model proves to be very effective when used in the HAM10000 dataset, there is a risk of possible domain changes when applied to the image obtained with the help of the other dermoscopy devices or the clinical environment. Variations in lighting, model generalization may depend upon imaging conditions, resolution, and other factors. Future research will involve the domain adaptation methodology and cross-dataset validation in order to enhance stability in the context of various clinical settings. Transformer-based models have shown good performance in medical image classification tasks because they can capture long-range dependencies and contextual relationships between images in the image itself that are not limited to the local context of a single pixel area (Li et al., 2024). Nevertheless, such architectures can be very computationally-intensive and demand large-scale training data. Conversely, the suggested CNN-BiLSTM model offers the middle ground as it offers the ability to extract features in an efficient manner and achieve sequential modeling at the same time. Through this hybrid architecture, the effective feature representation is achieved with a reasonably low complexity of computation. Moreover, according to the recent literature, it is indicated that large-scale datasets and solid model architectures are crucial to trustworthy clinical implementation of automated dermatology architecture systems (Al-Waisy et al., 2025). Further investigation of multimodal methods and transformer improvements might be considered in the future to achieve an even higher level of classification accuracy and generalization when used on a wide range of medical imaging data.

### 4.9 Comparison with state-of-the-art methods

When compared to the latest state-of-the-art approaches, it can be noted that our CNN-BiLSTM model is more accurate and recalls more melanomas with much fewer parameters. Most of the current methods depend on deep CNNs, transformers, or ensemble methods that involve numerous parameters (Agarwal and Mahto, 2025). The efficiency of our model is critical in order to apply it practically in clinical and teledermatology practice. The findings indicate that a properly implemented hybrid architecture is capable of being even more efficient than more complicated models and being lightweight, understandable, and computable. Table 5 shows the comparison of skin lesion classification SOTA methods. Even though the developed CNN-BiLSTM model has good performance, there are still some limitations on the HAM10000 dataset. First, the model was tested on one dataset, and cross-dataset validation would be done. Enhance the generalization assertions. Second, the dermoscopic image domain may be introduced by variations brought about by various acquisition devices and shift challenges. Further studies will be on domain adaptation procedures and multi-data analysis. Transformer-based models have also demonstrated good results in medical image classification including Vision Transformers that have been introduced recently. Nevertheless, the suggested CNN-BiLSTM model demonstrates competitive results and has a reduced computational complexity and better efficiency in dermoscopic images classification.

**Table 5:** Comparison of the proposed CNN-BiLSTM model with state-of-the-art skin lesion classification methods.

References	Method	Architecture model	Accuracy (%)	Melanoma recall (%)	Parameters (M)
Tschandl et al. (2018)	ResNet + BiLSTM	CNN-RNN Hybrid	97.6	95.1	32.4
Pacheco and Krohling (2020)	ResNet-50	Deep CNN	94.8	91.5	25.6
Reddy et al. (2023)	Vision transformer	Transformer	96.8	94.2	86.4
Oliveira et al. (2020)	VGG-19	Deep CNN	93.4	89.2	143.7
Shaik et al. (2025)	Multi-scale CNN	Multi-scale CNN	96.9	94.4	18.6
Padhy et al. (2025)	DenseNet-121	Deep CNN	95.2	92.1	8.0
Schuster and Paliwal (1997)	Attention CNN-LSTM	Hybrid + attention	97.2	93.8	15.2
Reis et al. (2022)	GAN + CNN ensemble	Hybrid + GAN	97.5	94.9	~50
Radman and Suandi (2021)	EfficientNet-B3	Deep CNN	96.1	93.7	12.0
**Proposed**	**Hybrid**	**CNN+BiLSTM**	**98.7**	**95.5**	**3.8**

Bold indicates the proposed method (CNN-BiLSTM).

## 5 Conclusion

A significant health issue of concern to the entire world is skin cancer, particularly melanoma, due to its aggressive nature and high fatality rate. Timely and correct diagnosis enhances the survival of the patient, yet classical methods, which depend on visual inspection and dermoscopy, are subjective and depend on the observer. To address these shortcomings, this paper presents a hybrid CNN-BiLSTM model of automated classification of melanoma and multi-class skin lesions based on the HAM10000 dataset. The framework integrates the convolutional neural networks in the hierarchical spatial feature extraction with the bidirectional long short-term memory networks in the full contextual modeling. The preprocessing pipeline consisted of a powerful noise removal mechanism starting with median filtering, lesion localization with adaptive thresholding, augmentation of the data, uniform scaling of the values through pixel normalization, and extraction of the feature by the ABCDE rule that is inherent to the clinical standards. This method enhances the quality of images and model prediction, with 97.2% precision. It is concerned with the pressing need to reduce the rate of missed diagnoses (false negatives) without producing too many false alarms. The system is appropriate as a computer-aided dermatological diagnostic tool in daily practice due to its lightweight architecture and rapid inference. Despite the positive performance of the framework, there are a number of limitations. It has been tested using the HAM10000 dataset only and should be tested on external and multi-institutional datasets to demonstrate its applicability to different groups of patients and imaging equipment. The existing implementation also lacks the use of patient metadata or longitudinal lesion tracking, which would additionally increase accuracy. Future studies are needed to confirm the framework on various data sets, the use of attention mechanisms and explainable AI tools such as Grad-CAM to improve interpretability, multimodal data such as patient clinical metadata, and prospective clinical trials to confirm the usefulness of diagnosing in clinics and patient outcomes. These measures will enhance the clinical feasibility of the framework and will make it easier to apply the framework in actual healthcare implementation.

## Data Availability

The original contributions presented in the study are included in the article/supplementary material, further inquiries can be directed to the corresponding author.
